# *Limosilactobacillus reuteri* Alleviates Anxiety-like Behavior and Intestinal Symptoms in Two Stressed Mouse Models

**DOI:** 10.3390/nu16183209

**Published:** 2024-09-22

**Authors:** Liang Zhang, Shuwen Zhang, Minzhi Jiang, Xue Ni, Mengxuan Du, He Jiang, Mingxia Bi, Yulin Wang, Chang Liu, Shuangjiang Liu

**Affiliations:** 1 State Key Laboratory of Microbial Technology, Shandong University, Qingdao 266237, China; 202190900070@sdu.edu.cn (L.Z.); 202000141061@mail.sdu.edu.cn (S.Z.); jiangminzhi2006@126.com (M.J.); nixue1140317984@foxmail.com (X.N.); dumengxuan2014@126.com (M.D.); jiangh@sdu.edu.cn (H.J.); bimx@sdu.edu.cn (M.B.); wangyulin@sdu.edu.cn (Y.W.); liu.c@sdu.edu.cn (C.L.); 2 State Key Laboratory of Microbial Resources, Institute of Microbiology, Chinese Academy of Sciences, Beijing 100101, China

**Keywords:** *Limosilactobacillus reuteri*, probiotics, strain-specific, anxiety-like behavior, gastrointestinal symptoms, gut microbiota

## Abstract

Background/Objectives: *Limosilactobacillus (Lm.) reuteri* is a widely utilized probiotic, recognized for its significant role in alleviating symptoms associated with gastrointestinal and psychiatric disorders. However, the effectiveness of *Lm. reuteri* is strain-specific, and its genetic diversity leads to significant differences in phenotypes among different strains. This study aims to identify potential probiotic strains by comparing the strain-specific characteristics of *Lm. reuteri* to better understand their efficacy and mechanisms in alleviating stress-induced anxiety-like behaviors and gastrointestinal symptoms. Methods: We cultivated 11 strains of *Lm. reuteri* from healthy human samples and conducted phenotypic and genomic characterizations. Two strains, WLR01 (=GOLDGUT-LR99) and WLR06, were screened as potential probiotics and were tested for their efficacy in alleviating anxiety-like behavior and intestinal symptoms in mouse models subjected to sleep deprivation (SD) and water avoidance stress (WAS). Results: The results showed that the selected strains effectively improved mouse behaviors, including cognitive impairment and inflammatory response, as well as improving anxiety and regulating gut microbiota composition. The improvements with WLR01 were associated with the regulation of the NLRP3 inflammasome pathway in the SD model mice and were associated with visceral hypersensitivity and intestinal integrity in the WAS model mice. Conclusions: In summary, this study identified the *Lm. reuteri* strain WLR01 as having the potential to alleviate anxiety-like behavior and intestinal symptoms through the analysis of *Lm. reuteri* genotypes and phenotypes, as well as validation in mouse models, thereby laying the foundation for future clinical applications.

## 1. Introduction

Chronic stresses have adverse effects on mental and body health. Long-term exposure to stress conditions can lead to endocrine disorders, immune imbalances (including inflammation), and dysbiosis of the gut microbiota, which exaggerates and triggers mental illnesses such as anxiety and depression [1,2,3]. Anxiety and depression are the most common mental illnesses, characterized by panic, weight loss, fatigue, and cognitive and motor dysfunction [4,5]. Currently, anxiety is becoming a global issue, especially in developing countries, where the prevalence of this disease is rapidly increasing. The estimated incidence rate exceeds 2.5% [6,7]. Although the understanding of anxiety has become increasingly profound in recent years, its exact physiological and pathological mechanisms are still unclear. The current treatment methods include chemical drugs or psychological therapy. Treatment with chemical drugs generally has certain side effects, and psychological therapy raises compliance issues and is not suitable for long-term use. Consequently, there is an urgent need to establish effective treatment methods that are free of side effects and to elucidate their mechanisms of action.

In recent years, probiotics have attracted increasing attention due to their wide clinical applications and beneficial health effects on various clinical diseases, including mental disorders and gastrointestinal diseases [8,9]. *Lm. reuteri*, as a commonly used probiotic, can regulate the host’s physiological and immune responses at multiple levels, with potential benefits such as inhibiting inflammation, improving intestinal barrier function, regulating immune responses, and maintaining intestinal microbiota symbiosis [10]. Meanwhile, *Lm. reuteri* also has a wide range of nutritional benefits and has been shown to enhance nutrient absorption [11]. One study indicated that *Lm. reuteri* can synthesize pseudovitamin B_12_, which is crucial for maintaining neurological health [12]. Specifically, *Lm. reuteri* NK33 was applied for the prevention and medication of anxiety/depression, where it reduced the levels of corticosterone, IL-6, and LPS in the blood and introduced the infiltration of Iba1^+^ and LPS^+^/CD11b^+^ cells (activated microglia) into the hippocampus [13]. Another strain, *Lm. reuteri* 8008, could alleviate anxiety/depression-like behaviors by regulating the gut microbiota in depressed mice [14]. These studies suggest that *Lm. reuteri* strains provide a wide range of nutritional, physiological, and psychological health benefits. Studies have also shown that not all *Lm. reuteri* strains exhibit probiotic effects. Zhang et al. found that strain FSCDJY33M3 effectively enhanced intestinal barrier function and modulated immune activity in mice, while strain FCQHCL8L6 showed no significant effects [15]. These studies indicate that the strain-specific effectiveness of *Lm. reuteri* may be related to the physiological and biochemical characteristics of the strains, which are governed by each strain’s genetic heterogeneity. Research has found that, compared to using *Lm. reuteri* alone, combination with four other probiotics significantly enhanced the effect in relieving constipation [16]. In addition, *Lm. reuteri* synergistically improved metabolic disorders with caffeoylquinic acid, but it had no effect when used alone [17]. These studies suggest that *Lm. reuteri* may have enhanced effects by synergistically interacting with other beneficial bacteria through its unique physiological and biochemical functions.

The current study aims to screen potential *Lm. reuteri* probiotic strains through comparative analysis of the *Lm. reuteri* genome and detection of their physiological and biochemical characteristics and to evaluate the regulatory effects of candidate strains on anxiety-like behavior and gastrointestinal symptoms using two chronic stress mouse models, as well as to explore their mechanisms of action. This research potentially offers a novel, natural approach to managing anxiety and gastrointestinal disorders.

## 2. Materials and Methods

### 2.1. Isolation, Identification, Genome-Sequencing, and Analysis of Bacterial Strains

Eleven *Lm. reuteri* strains were isolated from healthy Chinese volunteers and cultured in De Man, Rogosa, and Sharpe (MRS) medium (Qingdao Hope Bio-Technology Co., Ltd., Qingdao, China, containing L-cysteine at 0.05%). Strain WLR08, isolated from a commercial product, served as a reference for testing. All bacterial strains were preliminarily identified using 16S rRNA gene sequences by BLAST using http://blast.ncbi.nlm.nih.gov/Blast.cgi.

Whole-genome sequencing of *Lm. reuteri* strains was completed by Magigene Tech Co., Ltd. (Shenzhen, China) using the Illumina NovaSeq PE150 (Illumina, Inc., San Diego, USA) and ONT Nanopore (Oxford Nanopore Technologies, Oxford, UK) sequencing platforms. The coding sequence (CDS) in the genome was predicted using Glimmer (version 3.02) [18]. Prokka (version 1.14.6) and Roary (version 3.13.0) were used to calculate the pan-genome and core genome [19,20]. A phylogenetic tree was constructed using FastTree (version 2.1.11) [21] and visualized with the iTOL web tool (version 6.9) [22]. The ANI analysis was calculated using the average nucleotide identity based on orthologous genes (OrthoANI) [23]. The enzymes and genes involved in carbohydrate metabolism were annotated using the Carbohydrate Active Enzymes Database (CAZymes) (dbCAN HMMdb version 12.0) [24]. The Comprehensive Antibiotic Resistance Database (CARD) was used to search for drug-resistance-related genes [25] using an identity threshold of 50%. The genomic data for the strains in this study were deposited in the National Microbiology Data Center (NMDC) (https://nmdc.cn/resource/genomics/project/detail/NMDC10019034, accessed on 25 July 2024).

### 2.2. Phenotypic Characterization of Lm. reuteri Strains

The generation time and tests for pH and bile tolerance were determined as previously described [26,27]. Fresh bacterial cultures were inoculated into MRS medium and incubated anaerobically at 37 °C. Growth was monitored every 2 h using OD_600_ measurements, and the generation time was calculated based on the change in OD_600_. The generation time (where P0 and Pt represent the initial and final OD_600_ values of the bacterial cultures, respectively): *n* = (log Pt − log P0)/log2.

Acid and alkaline tolerances were tested in MRS medium and PBS buffer at pH 2.5, 3.5, 4, 9, and 10. After 24 h anaerobic incubation, cells were collected, resuspended in PBS, and incubated at 37 °C for 4 h. The survival rate was determined using the dilution plating method.

$$\left(\%\right)\;survival=\frac{log\;CFU\;cells\;survived}{log\;initial\;cells\;inoculated}\times 100\%$$

Oxgall bile (0.3%, *w*/*v*) was added to the MRS liquid medium for the experimental group, while the control group contained no oxgall bile. Both groups were inoculated with a 1% (*v*/*v*) seed culture of *Lm. reuteri*, and growth was monitored for changes in optical density at 600 nm (OD_600_) every 24 h.

The methods of carbohydrate utilization, production of short-chain fatty acids and other organic acids, and antibiotic susceptibility assays were measured as previously described [28]. The carbohydrate metabolism of *Lm. reuteri* was analyzed using BIOLOG ANI microplates (BIOLOG Inc., Hayward, CA, USA) with 95 substrates. Following the manufacturer’s instructions, the plates were incubated anaerobically at 37 °C for 24 h, and growth was assessed by measuring absorbance changes at 590 nm and 750 nm. Short-chain fatty acids (SCFAs) and organic acids were quantified using GC/MS after extracting the fermentation broth with ethyl acetate for SCFAs, followed by derivatization for organic acids. The extracted phase was analyzed using a DB-5MS capillary column (Thermo Fisher Scientific Inc., Waltham, MA, USA). Antibiotic susceptibility was tested via the disc diffusion method on MRS agar, with inhibition zones measured after 24 h of anaerobic incubation at 37 °C.

### 2.3. Animal and Treatment

The animal studies complied with the requirements of the Helsinki Declaration and were approved by the Ethics Committee of Shandong University for the care and use of laboratory animals (Approval No. SYDWLL-2023-063). Female and male wild-type C57BL/6J mice, aged five to seven weeks, were purchased from Beijing Vital River Laboratory Animal Technology Co., Ltd (Beijing, China). The housing temperature was maintained at 24 ± 2 °C with a humidity level of 60 ± 20%. All mice were provided with sterile water and a standard chow diet ad libitum. The mice were acclimated for one week before the experiment and then randomly selected and divided into groups (6 mice per group).

Bacterial strains: *Lm. reuteri* was cultured in MRS medium at 37 °C for 24 h, centrifuged at 4 °C (4000× *g*, 10 min), and washed twice with sterile PBS. The collected cells were then resuspended in PBS solution to obtain a bacterial suspension.

Experiment of SD: Mice were randomly divided into 5 groups (*n* = 6) according to the experimental requirements: control group, SD group, and *Lm. reuteri*-treated groups (WLR01 group, WLR06 group, and WLR08 group). In the 1st week, all mice underwent environmental adaptation using sleep deprivation devices. From the 2nd week to the 11th week, the three *Lm. reuteri*-treated groups were given oral gavage of strains WLR01, WLR06, and WLR08, respectively (0.1 mL, 2 × 10^9^ CFU per day per mouse), while the control and SD groups received equal volumes of PBS. Subsequently, all mice in the SD group and the *Lm. reuteri*-treated groups were subjected to sleep deprivation. They were deprived of sleep for 23 h and exposed to light interference each day, with only 1 h of rest.

Experiment of WAS: The mice were randomly assigned to 5 groups (*n* = 6) according to the treatment: control group, SD group, and *Lm. reuteri*-treated groups (WLR01 group, WLR06 group, and WLR08 group). From day 1 to day 7, all mice underwent environmental adaptation; from day 8 to day 22, the *Lm. reuteri*-treated groups were given oral gavage of strains WLR01, WLR06, and WLR08, respectively (0.1 mL, 2 × 10^9^ CFU per day per mouse), while the control and SD groups received equal volumes of PBS. Subsequently, according to the reported method, they received 1 h of water avoidance stress (WAS) treatment every day from day 13 to day 22. The number of feces in the container was counted at the end of each 1 h WAS treatment, and the autonomous regulation of colon movement was evaluated.

### 2.4. Behavioral Test and Visceral Sensitivity Assessment

The elevated plus maze (EPM) and Y-maze (including spontaneous alternation and novel arm recognition) were used to assess the mice’s anxiety-like behavior and recognition memory. Three days before the end of the experiment, the EPM and Y-maze experiments were conducted as described previously [29,30]. During the EPM experiment, the mice were recorded for arm entries and the time spent on open arms over a period of 6 min. In the Y-maze experiment, the mice were allowed to freely explore, and their spontaneous alternation rate was measured over a 5-min duration. In the novel arm recognition experiment, after exploring the Y-maze for 10 min as training, the mouse rested for 2 h. It then returned to its starting arm and was allowed to move freely for 5 min. The activity time and movement distance of the mouse in the novel arm were recorded.

After the last WAS treatment, mice were fasted for 18 h and then subjected to a colorectal distension test to evaluate visual sensitivity. The experimental method and evaluation standards refer to Chen et al. [31]. The colorectal distension (CRD) air pressures were set at 0.05, 0.1, 0.15, and 0.2 mL.

### 2.5. Collection of Feces, Blood, and Tissue Samples

On the day before the experiment concluded, fecal samples were collected from the mice under sterile conditions, quickly frozen in liquid nitrogen for half an hour, and then stored at −80 °C until used for gut microbiota analysis. At the end of the experiment, the mice were anesthetized with isoflurane, and blood was collected by enucleation. The blood samples were kept at room temperature until coagulation and then centrifuged at 3000 rpm for 10 min to collect the serum, which was stored at −80 °C. The colon tissue was divided into 1 cm segments and rinsed with pre-cooled PBS solution to remove the intestinal contents. A portion of the segments was fixed in 4% paraformaldehyde solution overnight for further analysis, while the remainder was quickly frozen in liquid nitrogen and stored at −80 °C for subsequent RNA extraction and analysis. The brain was rapidly removed and placed on a chilled dissection platform. The brain was carefully dissected to isolate the hippocampus, which was then quickly frozen in liquid nitrogen and stored at −80 °C for subsequent analysis.

### 2.6. Cytokine Concentration Analysis of the Serum and Histological Evaluation

To evaluate the changes in IL-6, TNF-α, IL-1β, MDA, CORT, and CRH levels in mouse serum, we used corresponding ELISA kits (Boshen Co., Ltd., Nanjing, China) following the manufacturer’s instructions for detection [32]. Colon tissue was fixed with 4% paraformaldehyde and embedded in paraffin. Antibodies against occludin (OCLN) and zonula occludens 1 (ZO-1) were used for immunohistochemistry (IHC). This part of the experiment was completed by Servicebio Technology Co., Ltd. (Wuhan, China).

### 2.7. Real-Time qPCR Analysis

Total RNA was extracted from colon tissues and hippocampal samples of each mouse using Trizol (Vazyme Biotech, Nanjing, China) solution for analysis at the gene transcription level according to Liu et al. [32]. The cDNA was synthesized using the HiScript III RT SuperMix (Vazyme Biotech, Nanjing, China). The relative mRNA expression level was normalized to the internal control GAPDH (glyceraldehyde-3-phosphate dehydrogenase) gene and calculated using the comparative threshold cycle (Ct) method [33]. Each qPCR reaction was performed in duplicate, and the expression level was determined as the mean value. Primer sequences are listed in Table 1.

### 2.8. Gut Microbiota Analysis

A fecal bacterial DNA extraction kit (Tiangen, Beijing, China) was used to extract total microbial DNA from fecal samples. The PCR technique was employed to amplify the V3–V4 region of the 16S rRNA gene. Sequencing of the gut microbiota was performed by the company (Benagen, Wuhan, China; Personalbio, Shanghai, China). The clean data were denoised (command: -unoise3) and analyzed using Qiime2 (version: 2023.7) [34]. Differences in gut microbiota composition between the groups were assessed using the least discriminant analysis effect size (LEfSe) method, implemented through the online tool OmicStudio (version 2.1) [35].

### 2.9. Statistical Analysis

All data are presented as the standard errors of the mean (SEM). A one-way analysis of variance (ANOVA) was used to analyze the results, and Tukey’s multiple comparison test was subsequently employed to determine the statistical significance. A *p*-value of ≤0.05 was considered to indicate statistical significance. All statistical evaluations, including box–whisker plots and bar graphs, were conducted using GraphPad Prism version 8 (GraphPad Software, Inc., La Jolla, CA, USA).

## 3. Results

### 3.1. Genomic Diversity of Lm. reuteri at the Strain Level and Association with Phenotypes

**Figure 1:**
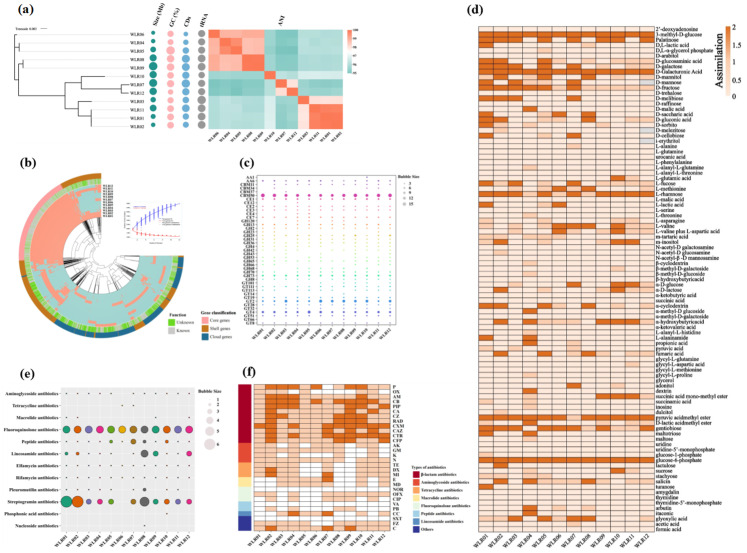
Comparative genome analysis and association of geno- and phenotypes of *Lm. reuteri* strains. (**a**) Phylogenetic tree constructed with the neighbor joining method based on core gene alignment (left). Genome features include genome sizes, GC%, CDs numbers, number of tRNA, and average nucleotide identity (ANI) (right). (**b**) Pan- and core-genome analysis showing the growing pan-gene numbers as genome numbers increase (top right); pan-genome analysis of *Lm. reuteri* strains. Each ring represents one strain, and each radial extension in the ring corresponds to a particular gene (red: present; blue: absent). The outer grey–green ring represents COGs functionality annotation, with grey indicating known and green indicating unknown functions. The outermost ring highlights particular gene collections: core, pink; shell genes, brown; and cloud genes, dark blue. (**c**) Genotypic and phenotypic analysis of carbohydrate utilization, genes related to carbohydrate activity enzymes. (**d**) BIOLOG test results for carbon sources assimilation. (**e**) Genome annotation for antibiotic resistance. (**f**) Experimental observation on bacterial resistance to 30 antibiotics (white = tolerant; faint yellow = intermediate; yellow = intolerant); the *Lm. reuteri* strain WLR08 was analyzed in parallel for reference. Abbreviations: P: penicillin; OX: oxacillin; AM: ampicillin; CB: carbenicillin; PIP: piperacillin; CA: cephalexin; CZ: cefazolin; RAD: cephradine; CXM: cefuroxime; CAZ: ceftazidime; CTR: ceftriaxone; CFP: cefoperazone; AK: amikacin; GM: gentamicin; K: kanamycin; N: neomycin; TE: tetracycline; DX: doxycycline; MI: minocycline; E: erythromycin; MD: medemcyin; NOR: norfloxacin; OFX: ofloxacin; CIP: ciprofloxacin; VA: vancomycin; PB: polymyxin B; CC: clindamycin; SXT: trimethoprim-sulfamethoxazole; FZ: furazolidone; C: chloramphenicol.

General genome features. In this study, 11 strains of *Lm. reuteri* were isolated and subjected to DNA sequencing. Genomic sequences were acquired for the 11 strains and a commercial probiotic *Lm. reuteri* strain (WLR08). Although all *Lm. reuteri* strains originated from healthy individuals, their genomes exhibited significant differences. The genome sizes of the 12 strains varied, ranging from 1.88 to 2.18 Mb, with tRNA gene copy numbers ranging from 69 to 71. The GC contents ranged from 38.82% to 39.18%. The constructed phylogenetic tree based on the core genome indicates that the 12 strains of *Lm. reuteri* developed into two evolutionary lineages (Figure 1a). We further compared the average nucleotide identity (ANI) values of the 12 *Lm. reuteri* genomes. The results showed that their ANI values ranged from 94.9% to 99% (Figure 1a), indicating the potential presence of a subspecies of *Lm. reuteri* among these 12 strains. In order to better understand the genomic functional differences of *Lm. reuteri*, whole-genome and core-genome analyses were conducted. In total, 5037 homologous genes were identified in *Lm. reuteri*, with 1188 homologous genes in the core genome, accounting for 23.6% of the total genome. There were a total of 3849 accessory genes, accounting for 76.4% of the total genome. The pan-genome is defined as the sum of all genes within a species. As shown in Figure 1b, the size of the pan-genome increased with the addition of more strains, while the number of core genes tended to stabilize. This suggests that *Lm. reuteri* exhibits both strain diversity and genetic diversity. Functional analysis of the core genes of *Lm. reuteri* showed that the core genome comprises genes associated with replication, transcription, translation, nucleotide metabolism, carbohydrate metabolism, amino acid metabolism, lipid metabolism, etc. Among them, carbohydrate-metabolism-related genes account for approximately 6.15% of the core functional genes, and amino acid metabolism-related genes account for 7.86%. However, 17.68% of the core genome functions are unknown (Figure 1b).

Association of carbohydrate utilization and genotypes. The CAZy database was used to predict the carbohydrate hydrolysis enzyme genes of each genome, evaluate the carbohydrate fermentation genes of *Lm. reuteri*, and validate the predicted carbohydrate hydrolysis ability through BIOLOG (Figure 1e,f). As shown in Figure 1e, the glycosidase genes of *Lm. reuteri* include 17 glycoside hydrolases (GHs), 12 glycosyltransferases (GTs), 4 carbohydrate-binding modules (CBMs), 6 carbohydrate esterases (CE), and 2 helper activity (AAs) genes. The GH genes are closely related to polysaccharide degradation. The genes with the highest number in the GH family are GH13 and GH73. The most abundant enzymes are members of the GH family, followed by the GT family (Figure 1e). Among the 12 genomes, there are a total of 41 genes encoding carbohydrate active enzymes, such as GT4 (sucrose synthase) and GH13 (α- amylase).

The BIOLOG results revealed inconsistencies between various genotypes and phenotypes. For instance, strains with the GH13 gene were unable to degrade D-melibiose. Additionally, we assessed the utilization of amino acids and organic acids by different strains of *Lm. reuteri.* The findings indicated variations in their utilization efficiency (Figure 1f), highlighting the metabolic diversity among *Lm. reuteri* strains.

Association of antibiotic resistance phenotypes and genotypes. Using the CARD database to estimate the resistance of the *Lm. reuteri* genomes, it was revealed that the resistance types of the 12 strains ranged from 9 to 22 (similarity > 50%), with slight differences among the strains (Figure 1c). Fluoroquinolone antibiotic genes were present in all strains. Compared with the other strains, WLR01 contains more genes for streptomycin antibiotics.

To further confirm the resistance of *Lm. reuteri* to 30 antibiotics, drug sensitivity experiments were conducted using the K-B method [36]. The results showed that the *Lm. reuteri* strains are sensitive to cephalosporin and chloramphenicol (Figure 1d) and resistant to vancomycin, indicating a significant difference in the sensitivity of *Lm. reuteri* to antibiotics. All 12 strains have genes for macrolide antibiotics, but WLR01, WLR08, and others are sensitive to erythromycin, indicating that gene prediction cannot fully represent their resistance.

### 3.2. Growth Rates, Tolerances to Bile Salt and Acidity/Alkalinity, and Production of Short-Chain Fatty Acids

**Figure 2:**
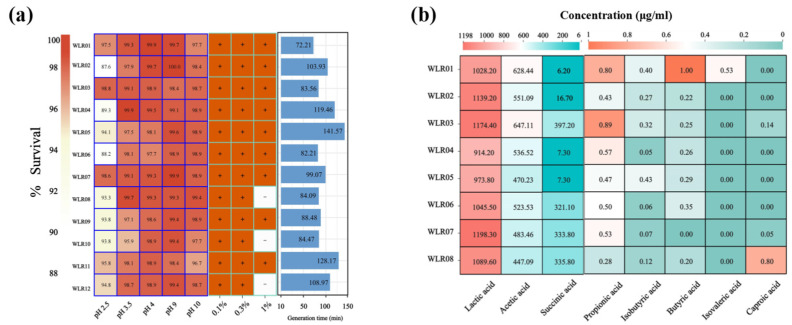
Biological characteristics of *Lm. reuteri* strains. (**a**) Evaluation of tolerance to acidity (pH 2.5, 3.5, and 4) and alkalinity (pH 9 and 10) (left); bile salt tolerance results (middle), following strains’ growth at different bile salt concentrations relative to the control group without bile salts (+: normal growth; −: delay of growth between the control and oxgall bile cultures); and germination rate of spores of *Lm. reuteri* isolates (right). (**b**) Production of SCFAs (acetic acid, propionic acid, isobutyric acid, butyric acid, isovaleric acid, and caproic acid) and other organic acids (lactic acid and succinic acid) by each of the 8 strains.

The strains’ growth rates (generation time), pH and bile tolerance, and production of short-chain fatty acids (SCFAs) and organic acids were assessed to evaluate their probiotic properties. All the *Lm. reuteri* strains exhibited significant differences. As shown in Figure 2a, under the condition of pH 2.5, all strains exhibited a survival rate above 85%. Specifically, four strains (WLR01, WLR03, WLR07, and WLR11) had survival rates > 95%. All strains maintained a high survival rate at lower pH. Compared with the low pH, all 12 strains exhibited excellent tolerance to higher pH. Even at pH 10, the survival rates of all strains were higher than 95%. Additionally, all 12 *Lm. reuteri* strains were resistant to 0.5% bile salt. The growth rates of the strains varied, with generation times ranging from 72 to 141 min. Among them, strains WLR01 and WLR06 were the top two growing strains, with generation times of 72 and 82 min, respectively (Figure 2a).

We evaluated the ability of eight strains of *Lm. reuteri* to produce SCFAs and other organic acids in vitro using the GC/MS system. The results showed that acetic acid and lactic acid were the main acids, with strain WLR03 exhibiting the highest acetic acid production at 647 μg/mL (Figure 2b). Short-chain fatty acids (SCFAs) such as propionic acid, butyric acid, isobutyric acid, and isovaleric acid were detected at low concentrations, ranging from 0 to 1 μg/mL. Notably, strain WLR01 exhibited the highest butyric acid production. Uniquely, only strain WLR01 could produce isovaleric acid. There was a significant difference in succinic acid production among the different strains, ranging from 6.2 to 397.2 μg/mL.

After conducting the comparative genomics and physiological analysis, WLR01, WLR06, and the reference strain WLR08 were selected for animal experiments based on their phylogenetic differences from the other strains and their physiological characteristics such as faster growing, stronger acid-base tolerance, and higher acid production capacity.

### 3.3. Chronic Stresses Induce Extensive Behavioral and Physiological Responses and Gut Microbiota Changes in Mice

**Figure 3:**
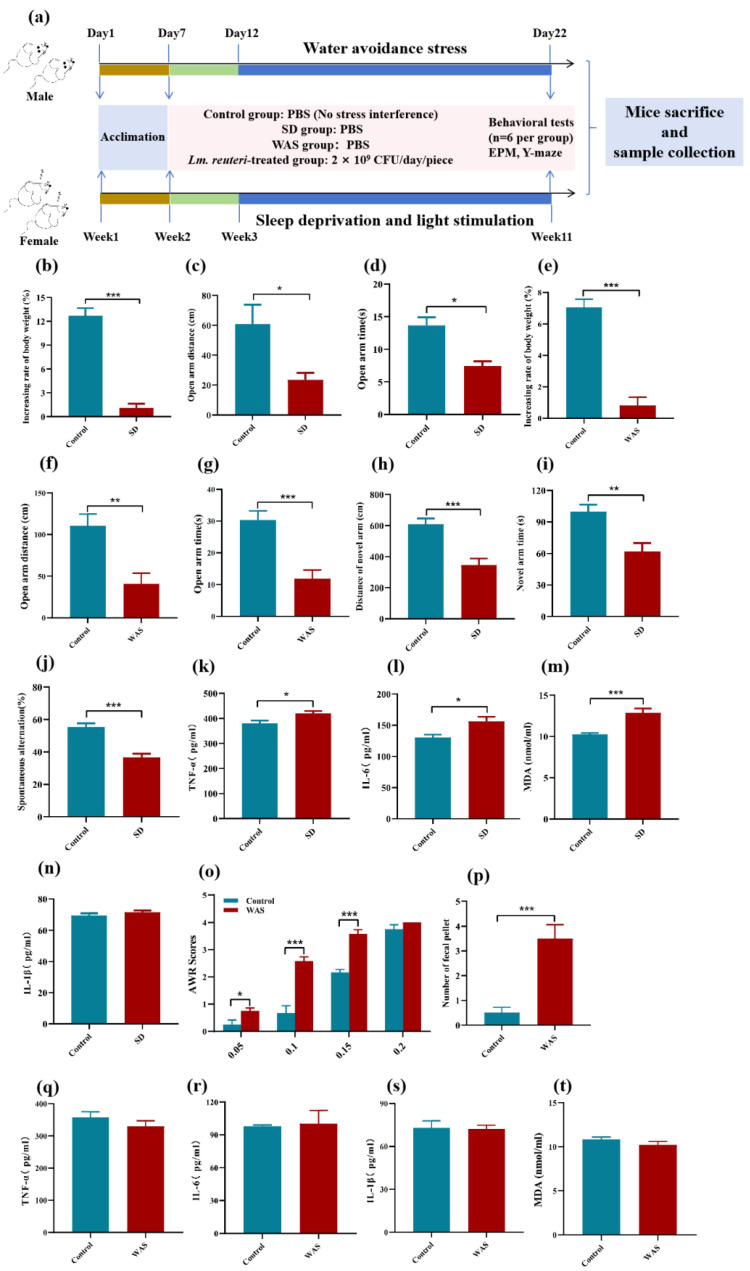
Chronic stresses induce extensive behavioral and inflammatory factors responses in mice (*n* = 6/group). (**a**) Schematic representation of the experimental design. (**b**) Percentage of weight gain during SD on the last day. (**c**) Distance of motion in open arms. (**d**) The time spent in open arms in SD mice. (**e**) Percentage of weight gain during WAS on the last day. (**f**) Distance of motion in open arms. (**g**) The time spent in open arms in WAS mice. (**h**) Distance of motion in novel arm. (**i**) The time spent in novel arms. (**j**) Spontaneous alternation rate. ELISA analyses of (**k**) TNF-α, (**l**) IL-6, (**m**) MDA, and (**n**) IL-1β in SD mice. (**o**) Abdominal withdrawal reflex scores in response to CRD. (**p**) The numbers of fecal pellets found in containers during WAS on the last day. ELISA analyses of (**q**) TNF-α, (**r**) IL-6, (**s**) IL-1β, and (**t**) MDA in WAS mice. Statistics: * *p* < 0.05, ** *p* < 0.01, *** *p* < 0.001.

Anxiety and body weight changes in mice. A schematic diagram for the experiment is illustrated in Figure 3a. To determine the effects of chronic stress on mice, we evaluated changes in the autonomous activity behavior and weight of mice after 12 weeks of SD and 12 days of WAS. Compared to the control group, the SD group of mice demonstrated reduced weight gain (Figure 3b). In the EPM experiment, we found that the movement distance of the SD group mice in the open arm (Figure 3c) and the residence time in the open arm (Figure 3d) were significantly reduced (*p* < 0.05). During the WAS experiment, the weight gain rate of the WAS group mice was significantly lower than that of the control group (Figure 3e). Compared to the control group, the movement distance (Figure 3f) and residence time (Figure 3g) of the open arm mice were both significantly reduced (*p* < 0.05). These results indicate that SD and WAS can induce anxiety-like behavior in mice and delay their weight gain.

Memory and cognitive function, intestinal symptoms, and expression of inflammatory factors in mice. We characterized the effects of chronic stress-induced anxiety on memory and cognitive function, intestinal symptoms, and inflammatory factors using Y-maze and ELISA methods. In the novel arm recognition experiment, it was observed that the SD group mice exhibited significantly reduced movement distances (Figure 3h) and time spent in the new arm (Figure 3i) compared to the control group. In the Y-maze spontaneous alternation experiment, the spontaneous alternation rate of the SD group mice was notably lower than that of the control group (Figure 3j). The results demonstrated an overexpression of pro-inflammatory cytokines (TNF-α and IL-6) and oxidative stress factors (MDA) in the serum of mice in the SD group (Figure 3k–n).

The colorectal dilation (CRD) test and fecal excretion rate were used to evaluate the effects of WAS-induced anxiety on visceral sensitivity and intestinal motility. Compared with the control group, the visceral sensitivity of mice in the WAS group was significantly increased (Figure 3o). Fecal pellet output numbers were significantly higher in WAS mice than in the control group (Figure 3p). However, the pro-inflammatory cytokines (TNF-α, IL-6, and IL-1β) and oxidative stress factors (MDA) did not show significant changes in the WAS group mice (Figure 3q–t).

**Figure 4:**
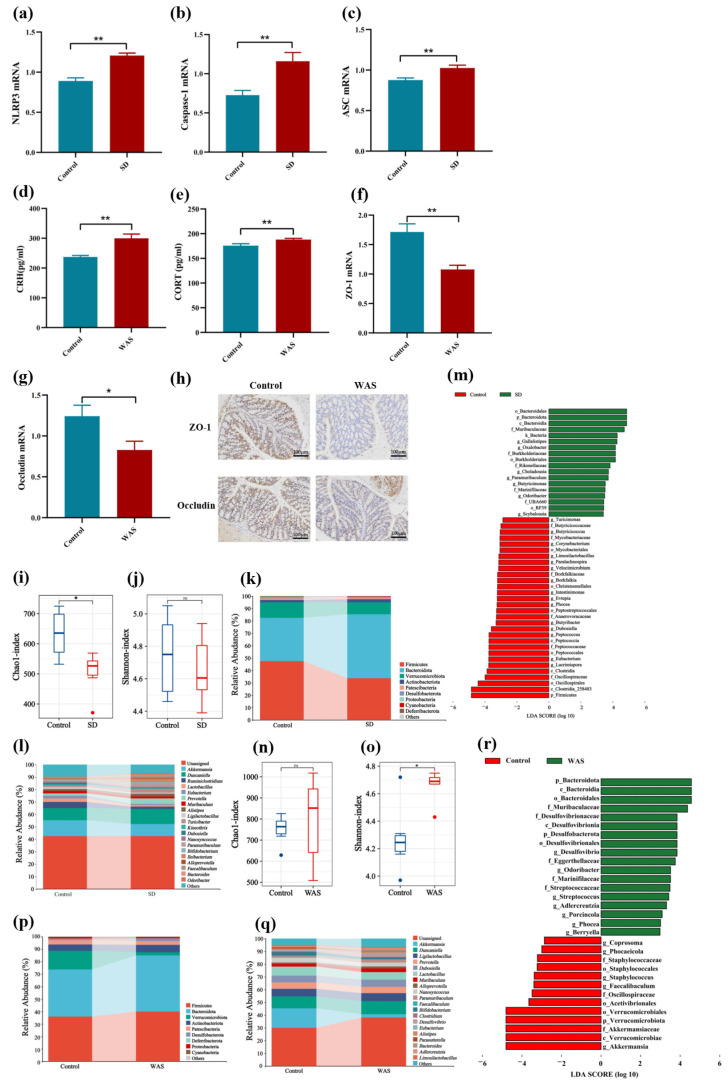
Chronic stresses induce physiological responses (NLRP3 inflammasome, HPA axis, and intestinal mucosal barrier) and gut microbiota dysbiosis in mice (*n* = 6/group). RT-qPCR of hippocampal caspase-1 mRNA normalized to expression of (**a**) NLRP3, (**b**) Caspase-1, and (**c**) ASC. The hypothalamic–pituitary–adrenal axis index of (**d**) CRH, (**e**) CORT, (**f**) colonic ZO-1 mRNA, and (**g**) occludin, normalized to expression of GAPDH. (**h**) Immunohistochemistry of intestinal tissue based on ZO-1 and occludin protein. (**i**,**j**) Chao1 index and Shannon index of fecal microbiota in SD mice. (**k**) Microbial taxa at the phylum level in SD mice. (**l**) The top 15 microbial taxonomic groups at the genus level in SD mice. (**m**) Distribution histogram based on LDA (LDA > 2) in SD mice. (**n**,**o**) Chao1 index and Shannon index of fecal microbiota in WAS mice. (**p**) Microbial taxa at the phylum level in WAS mice. (**q**) The top 15 microbial taxonomic groups at the genus level in WAS mice. (**r**) Distribution histogram based on LDA (LDA > 2) in WAS mice. Statistics: ns: not significant (*p* > 0.05), * *p* < 0.05, ** *p* < 0.01.

Activation of NLRP3 inflammasome and HPA axis and disruption of the intestinal barrier and gut microbiota structure in mice. Based on the aforementioned observations, in order to explore the changes in the NLRP3 inflammasome pathway in the hippocampus of mice showing SD-induced anxiety-like behavior, RT-qPCR was utilized to detect the mRNA expression levels of the key factors NLRP3, ASC, and caspase-1 in the NLRP3 inflammasome pathway. It was found that the relative expression levels of NLRP3, ASC, and caspase-1 in the SD group mice were significantly increased (Figure 4a–c). The results showed that SD could activate the NLRP3 inflammasome in the hippocampus.

Similarly, we detected changes in intestinal barrier function and serum HPA axis hormones in mice exhibiting anxiety-like behavior induced by WAS. The results of HPA axis hormones in serum are shown in Figure 4d,e. The levels of serum corticotropin-releasing hormone (CRH) and corticosterone (CORT) were significantly increased in the WAS group of mice (Figure 4d,e). RT-qPCR was used to detect the mRNA expression levels of the key factors ZO-1 and occludin in the colon. The results showed a decrease in the mRNA expression of occludin and ZO-1 in the colon of mice in the WAS group (Figure 4f,g). The immunohistochemical results also indicated a decrease in the expression levels of ZO-1 and occludin (Figure 4h). These findings suggest that the intestinal mucosal barrier function in mice was compromised.

The gut microbiota plays an important role in maintaining intestinal homeostasis, and dysbiosis of the gut ecosystem can result in immune dysfunction, chronic inflammation, and the development of various diseases. Therefore, we utilized 16S rRNA sequencing to investigate the effects of chronic stress (including SD and WAS) on the gut microbiota. In comparison to the control group, the Chao1 index and Shannon index revealed a notable decrease in the richness and evenness of the gut microbiota in the SD group (Figure 4i,j).

Furthermore, we analyzed the relative abundance of the gut microbiota at various taxonomic levels. The top 10 microbial communities at the phylum level with relative abundance are depicted in Figure 4k. Compared with the control group, the SD group showed a decrease in Firmicutes and an increase in Bacteroidetes. The 15 most abundant microbial communities at the genus level are shown in Figure 4l. In comparison to the control group, the relative abundance of *Prevotella*, *Alistipes*, and *Turicibacter* significantly increased in the SD group, while the abundance of *Akkermansia* and *Lactobacillus* decreased significantly (Figure 4l). In addition, differences in the abundance of taxa were assessed using linear discriminant analysis effect sizes (LEfSe). Based on linear discriminant analysis (LDA) > 2, the results indicated an increase in the abundance of *Odoribacter* in the SD group (Figure 4m).

We also investigated the effect of WAS on the gut microbiota and found no significant difference in Chao1 index between the control group and the WAS group. However, the Shannon index was significantly lower in the control group (Figure 4n,o). These results indicate that WAS induced changes in the gut microbiota diversity. As shown in Figure 4p, the phyla Firmicutes and Bacteroidetes had the highest relative abundance, and the proportion of Firmicutes and Bacteroidetes in the WAS group mice was significantly reduced.

At the genus level, we observed that *Akkermansia* and *Duncaniella* exhibited a higher relative abundance (Figure 4q). The LEfSe analysis showed that, compared with the control group, the relative abundance of certain bacterial genera in the gut microbiota of WAS mice, such as *Akkermansia*, was significantly reduced. In addition, compared to the control group, a rapid increase in the relative abundance of harmful bacterial genera, such as Desulfovibrio, was observed in the WAS group (Figure 4r).

This suggests that anxiety-like behavior induced by WAS can result in damage to the intestinal mucosa, activation of the HPA axis, and dysbiosis of the gut microbiota.

### 3.4. Lm. reuteri Alleviates Anxiety-like Behavior and Improves Physiological Indices in Stressed Mice

**Figure 5:**
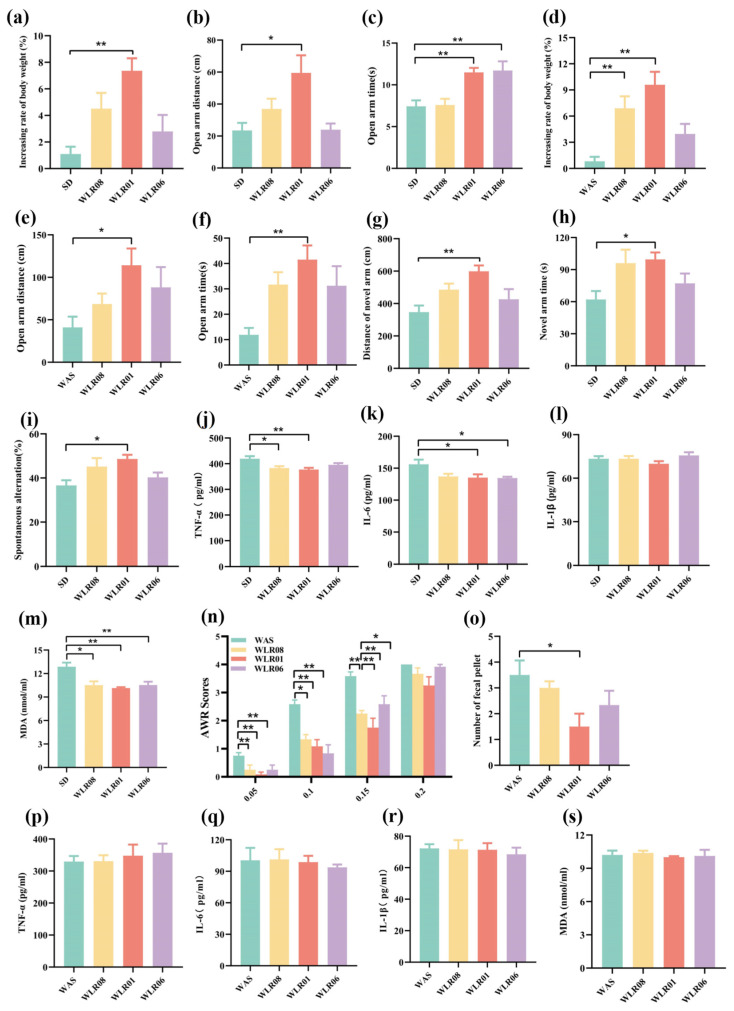
*Lm. reuteri* alleviates extensive behavioral and inflammatory factor responses in chronic stressed mice (*n* = 6/group). (**a**) Percentage of weight gain on the last day in SD mice. (**b**) Distance of motion in open arms. (**c**) The time spent in open arms. (**d**) Percentage of weight gain on the last day in WAS mice. (**e**) Distance of motion in open arms. (**f**) The time spent in open arms. (**g**) Distance of motion in novel arm. (**h**) The time spent in novel arms. (**i**) Spontaneous alternation rate. ELISA analyses of (**j**) TNF-α, (**k**) IL-6, (**l**) IL-1β, and (**m**) MDA in SD mice. (**n**) Abdominal withdrawal reflex scores in response to CRD. (**o**) The numbers of fecal pellet found in containers during WAS on the last day. ELISA analyses of (**p**) TNF-α, (**q**) IL-6, (**r**) IL-1β, and (**s**) MDA in WAS mice. Statistics: * *p* < 0.05, ** *p* < 0.01.

*Lm. reuteri* improves anxiety-like behavior and body weight gain. Our findings show that chronic stress, including SD and WAS, can affect weight gain in mice and induce anxiety-like behavior. In the SD experiment, the weight gain of mice after oral administration of *Lm. reuteri* (strain WLR01, WLR06, or WLR08) was restored to varying degrees (Figure 5a), and the movement distance and residence time of mice in the open arm increased (Figure 5b,c). Compared to the WAS group mice, *Lm. reuteri* treatment led to the recovery of body weight gain (Figure 5d), as well as an increase in open arm movement distance (Figure 5e) and residence time (Figure 5f), with strain WLR01 showing the most significant effect.

*Lm. reuteri* improves memory and cognitive function, intestinal symptoms, and expression of inflammatory factors in mice. Through the Y-maze new arm recognition experiment, we found that *Lm. reuteri* intervention in SD and WAS mice can increase the movement distance and residence time of mice in the new arm. In the Y-maze experiment, it was found that the spontaneous alternation rate of mice treated with *Lm. reuteri* intervention was higher than that of mice treated with SD or WAS, with WLR01 intervention showing a significant effect (Figure 5g–i). Additionally, we found that *Lm. reuteri* can inhibit the overexpression of TNF-α, IL-6, MDA, and other cytokines, essentially restoring them to the levels observed in the control group (Figure 5j–m). Although WLR06 and WLR08 could also inhibit the expression of these cytokines, their effects were weaker than those of WLR01.

The *Lm. reuteri* strain treatment significantly reduced visceral sensitivity in WAS mice (*p* < 0.05), with the WLR01 strain showing the most effectiveness (Figure 5n). As mentioned earlier, fecal pellet output numbers were significantly higher in WAS mice compared to the control group. The WLR01 treatment also notably decreased fecal pellet output numbers compared to those in the WAS group (*p* < 0.05) (Figure 5o). Intervention with the *Lm. reuteri* strains did not significantly affect the expression of inflammatory factors in WAS mice (Figure 5p–s).

These results suggest that *Lm. reuteri* strains alleviate memory and cognitive impairment and inhibit the expression of inflammatory factors in mice.

### 3.5. Lm. reuteri Functions via the NLRP3 Pathway, Improves Intestinal Mucosal Barrier Function, and Regulates HPA Axis in Chronic Stress-Induced Anxiety Mice

**Figure 6:**
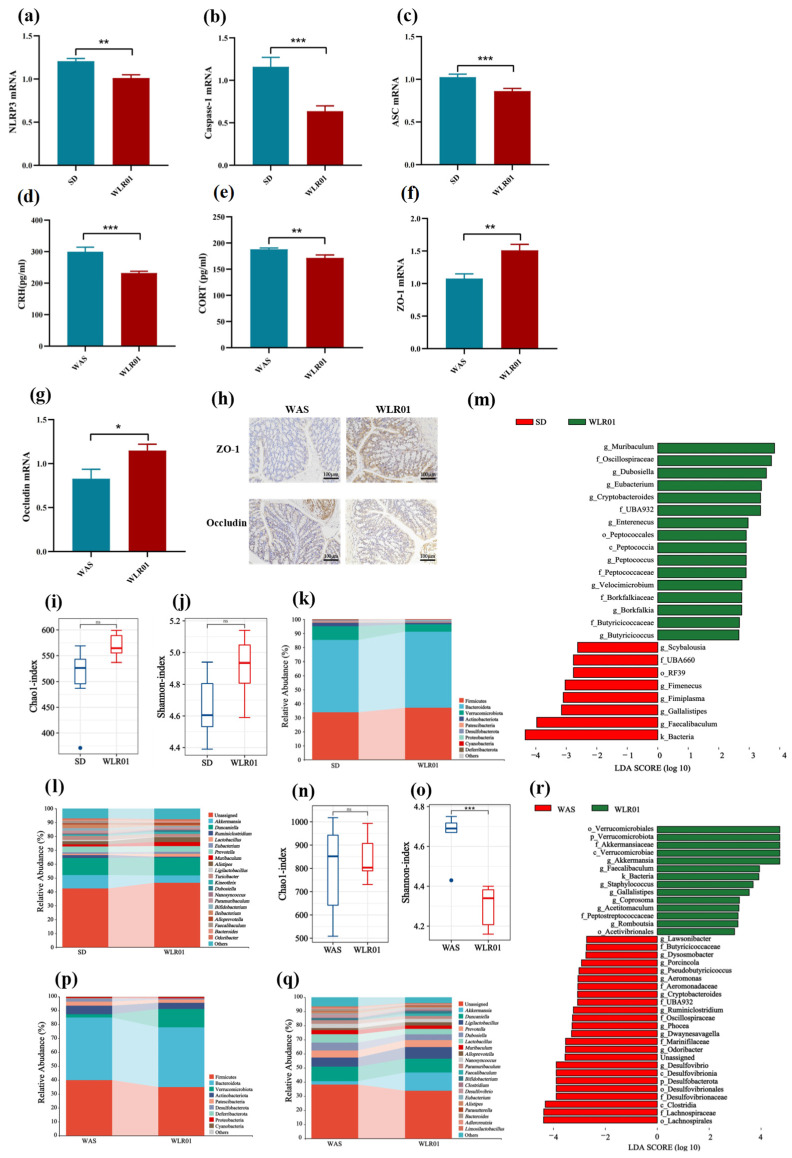
*Lm. reuteri* improved physiological responses (NLRP3 inflammasome, HPA axis, and intestinal mucosal barrier) and gut microbiota dysbiosis in chronically stressed mice (*n* = 6/group). RT-qPCR of hippocampal (**a**) NLRP3, (**b**) Caspase-1, and (**c**) ASC mRNA normalized to expression of GAPDH. The hypothalamic–pituitary–adrenal axis index of (**d**) CRH and (**e**) CORT. RT-qPCR of (**f**) colonic ZO-1 mRNA and (**g**) occludin, normalized to expression of GAPDH. (**h**) Immunohistochemistry of intestinal tissue based on ZO-1 and occludin protein in WAS mice. (**i**,**j**) Chao1 index and Shannon index of fecal microbiota in SD mice. (**k**) Microbial taxa at the phylum level in SD mice. (**l**) The top 15 microbial taxonomic groups at the genus level in SD mice. (**m**) Distribution histogram based on LDA (LDA > 2) in SD mice. (**n**,**o**) Chao1 index and Shannon index of fecal microbiota in WAS mice. (**p**) Microbial taxa at the phylum level in WAS mice. (**q**) The top 15 microbial taxonomic groups at the genus level in WAS mice. (**r**) Distribution histogram based on LDA (LDA > 2) in WAS mice. Statistics: ns: not significant (*p* > 0.05), * *p* < 0.05, ** *p* < 0.01, *** *p* < 0.001.

Compared with the SD group, the relative expression levels of NLRP3, ASC, and caspase-1 were significantly reduced after treatment with strain WLR01 (Figure 6a–c). *Lm. reuteri* WLR01 may reduce the inflammatory response of neural tissue and improve the memory cognitive ability of mice with anxiety-like behavior by inhibiting the NLRP3 inflammatory pathway in the hippocampus.

Strain WLR01 could prevent the elevation of serum CORT and CRH in WAS mice (Figure 6d,e). These results indicate that WLR01 treatment could prevent the altered hormone concentrations of the HPA axis in serum caused by WAS.

However, the levels of occludin and ZO-1 in mice significantly recovered after supplementation with *Lm. reuteri* WLR01 (Figure 6f,g), consistent with the results of the immunohistochemical analysis (Figure 6h). These findings suggest that the strain WLR01 can assist the host in restoring the intestinal barrier damage caused by WAS.

*Lm. reuteri* WLR01 regulates the gut microbiota structure. When investigating the effect of strain WLR01 on chronic stress-induced changes in the mouse gut microbiota, we observed that after being treated with strain WLR01, the Chao1 and Shannon indices increased in the WLR01 group compared to the SD group (Figure 6i,j). Our research discovered that treatment with strain WLR01 reversed the decrease in gut microbiota diversity caused by SD in mice. By comparing different taxonomic levels of species, we found that WLR01 treatment significantly increased the relative abundance of Firmicutes (Figure 6k). At the genus level, the abundance of *Lactobacillus* was restored after treatment with strain WLR01 (Figure 6l). Based on the linear discriminant analysis (LDA) > 2.5, the results indicated that the relative abundance of *Dubosiella* and *Muribaculum* in the WLR01-treated group significantly increased (Figure 6m).

We also investigated the effect of strain WLR01 on the gut microbiota of WAS mice and found that the Chao1 index did not significantly differ between the WAS group and the WLR01 treatment group (Figure 6n). However, the Shannon index reduced after treatment with strain WLR01 (Figure 6o). At the phylum level, it was observed that the relative abundance of Bacteroidetes decreased (Figure 6p). At the genus level, following treatment with strain WLR01, the relative abundance of certain probiotic genera, such as *Akkermansia* and *Bifidobacterium*, was significantly upregulated compared to the WAS group (Figure 6q). Based on the linear discriminant analysis (LDA) > 2, the results indicated that strain WLR01 significantly restored the relative abundance of *Faecalibaculum* and *Akkermansia* (Figure 6r).

## 4. Discussion

*Lm. reuteri* is one of the most common probiotic species and is found in various body sites, such as the gastrointestinal tract, urinary tract, skin, and breast milk [11]. The potential of *Lm. reuteri* for treating various diseases, including mental disorders, intestinal inflammation, and metabolic syndrome, has garnered increasing attention [37,38,39]. However, more studies are required to clarify the differences in the mechanisms and guide the development of probiotic products that can contribute to human health and well-being. Due to the heterogeneity of the strains, different strains are functionally polymorphic. For example, supplementation of *Lm. reuteri* ATCC-PTA-6475 can reverse social deficits in mice, but strain DSM 17938 cannot reverse social deficits in ASD mouse models [40]. Therefore, identifying specific functional probiotics through in vitro screening and animal models will facilitate future clinical applications. Published data indicate that integrating genotype and phenotype studies can significantly improve the screening of strains adapted to specific functions and applications [41]. In this study, we aimed to discover new resources of *Lm. reuteri* strains with alleviating effects on anxiety based on genomic and physiological characteristics. We also sought to explore the underlying mechanisms through which these strains exert their effects. The genome sizes of the 12 strains of *Lm. reuteri* were between 1.88 and 2.18 Mb, and their GC contents varied between 38.82% and 39.18%, which is consistent with the results of Yu et al. [42]. In addition, *Lm. reuteri* strains have different numbers of carbohydrate active enzyme genes in their genomes, including the abundant GH13, GH73, GT2, and GT4. The GH13 family can encode α-amylase. Previous research has found that α-amylase can convert glycogen into resistant glycogen, thereby inhibiting intestinal inflammation by increasing the expression of heme oxygenase-1 in mice [43,44]. Our study found that the amount of GH13 in strain WLR01 is higher than in other strains. These results demonstrate the genetic and functional diversity of *Lm. reuteri*, contributing to the understanding of the probiotic and biotechnological potentials of *Lm. reuteri*.

In this study, strains WLR06 and WLR01 showed excellent growth rates, acid-base tolerance, and SCFA production abilities. Thus, they were selected for further studies. According to research by Chen et al. [45], variations in genomic information among different strains may result in variances in physiological and biochemical characteristics. The physiological and biochemical characteristics of the strains, such as acid and alkali resistance and acid production ability, are important for the roles of the strains in vivo. The growth rate of bacteria may affect the effectiveness of probiotics, with faster growth rates having advantages for bacterial colonization and inhibition of other microorganisms [46]. Excellent tolerance to low pH and bile salts is essential for potential probiotics to reach the intestines alive and establish intestinal colonization, allowing them to perform functions such as supporting the intestinal barrier [47,48]. Short-chain fatty acids can regulate brain health and behavior through the immune system, induce T cell differentiation, control the production of inflammatory cytokines, affect the production of serotonin and other neurotransmitters, and have beneficial anti-inflammatory and mental health effects [49]. The ability to degrade multiple complex carbohydrates is one of the key genetic strategies that ensures the successful colonization and survival of bacteria in the intestinal environment [50]. The degradation of complex polysaccharides by probiotics helps other probiotics that depend on simple sugar growth in maintaining the intestinal balance and producing beneficial effects on the host. The results of this study showed significant differences in growth characteristics, acidity and bile acid tolerances, SCFA production ability, organic acid production ability, carbohydrate utilization ability, and antibacterial ability among the bacterial strains.

As previously reported, chronic stress may lead to anxiety-like behavior, slow weight gain, changes in bowel habits, and visceral hypersensitivity in mice [30,51]. This study utilized SD and WAS conditions for the mice and observed that, compared with the control group, the SD and WAS group mice exhibited slower weight gain, increased frequency of defecation, anxiety-like behavior, and visceral hypersensitivity. Research has shown that hyperthyroidism of the hypothalamic–pituitary–adrenal (HPA) axis is a cause of anxiety-like behavior and visceral hypersensitivity [52,53]. In this study, it was found that the WLR01 strain can reduce the secretion and release of CORT and CRH in mice, indicating that WLR01 may alleviate anxiety-like behavior and visceral hypersensitivity in WAS mice through the HPA axis. The intestinal mucus layer and epithelial cells, as the physical barrier of the intestine, play an important role in maintaining intestinal health by resisting harmful substances. Our study found that WLR01 treatment increased the expression levels of occludin and ZO-1 in mice, which is consistent with *Lactobacillus plantus* LZU-J-TSL6 being used as a probiotic to exert an indirect or direct anti-anxiety effect by protecting intestinal barrier integrity [54].

Studies have found that SD can induce oxidative stress in the brains of mice, thereby promoting inflammation in the brain and body and altering the gut–brain axis, leading to brain damage. These changes can result in a decline in cognitive function in mice [55]. In this study, it was observed that the motor coordination ability and cognitive levels of the mice in the SD group were significantly decreased. After treatment with strain WLR01, the motor ability and cognitive level of the mice were partially restored. Other studies [56] suggested that regulating cytokine levels can help hosts alleviate diseases; for example, the TNF-α antagonist infliximab is currently used as a treatment for autoimmune diseases but is also being considered as a treatment for drug-resistant anxiety. Additionally, it has been reported that an IL-1 receptor antagonist can reduce anxiety-related behavior in mice [57]. In this study, strain WLR01 was found to significantly downregulate the levels of the pro-inflammatory cytokines TNF-α and IL-6, as well as the oxidative stress factor MDA. These results suggest that WLR01 can exert a certain inhibitory effect on pro-inflammatory cytokines in mice, potentially alleviating symptoms associated with anxiety-like behavior by inhibiting the secretion of inflammatory and oxidative stress factors. 

Recent studies have shown that the interaction between inflammatory pathways and the neuroendocrine system is associated with symptoms such as pain, fatigue, memory impairment, and anxiety [58]. Studies have found that NLRP3 activation is essential to depression and fatigue pathogenesis, and NLRP3/caspase-1 inhibition therapy may be a promising option for treatment [57,59]. In our study, we found that the motor ability of the mice in the SD group was decreased. However, after treatment with WLR01, their motor ability improved. It was discovered that strain WLR01 could inhibit the abnormal activation of the NLRP3 inflammasome pathway. The expression levels of genes such as caspase-1 were significantly downregulated, suggesting that strain WLR01 might alleviate anxiety symptoms in SD mice by suppressing the activation of the NLRP3/caspase-1 pathway.

Although the pathological mechanism of anxiety-like behavior caused by chronic stress is not clear, the gut microbiota plays an important role in its occurrence and development. According to previous reports by Francino et al. [60], the gut microbiota is an important factor in regulating inflammatory tone, intestinal integrity, and function. The differences in gut microbiota between the chronically stressed population and healthy individuals are primarily attributed to variations in the abundance and diversity of specific bacterial types. Our study found that the WAS group showed an increase in microbial diversity compared to the control group and the group treated with WLR01, which is inconsistent with previous research [53]. We believe that specific types of microbiota are more important than gut microbiota diversity. Studies have shown that elevated levels of *Faecalibacterium* have the ability to produce butyrate, promote T cell differentiation, and reduce intestinal inflammatory responses, making it a promising candidate for probiotics [61]. Bacteria such as *Odoribacter* can disrupt the intestinal barrier. We identified that WLR01 reduced the abundance of genera such as *Odoribacter* and *Desulfovibrio* and upregulated the relative abundance of *Faecalibacterium* and *Akkermansia*. Some studies have also found that SD can disrupt the balance of the gut microbiota, and this imbalance may be related to the occurrence of anxiety-like behavior [62]. In this study, we observed a reduction in α-diversity within the gut microbiota of SD mice compared to healthy controls. WLR01 not only regulated the diversity level of the mouse microbiota but also made its intestinal microbiota structure more similar to that of the control group. At the genus level, it was found that the abundance of *Duncaniella* and *Prevotella* in the SD group mice was upregulated, which may be the key microbiota causing intestinal or in vivo inflammation. Simultaneously, WLR01 significantly promoted the upregulation of the abundance of *Muribaculum*. Previous studies found that *Muribaculum* is an important mucin monosaccharide forager, contributing to the reduction in intestinal inflammation [63]. These results suggest that WLR01 may play a role in alleviating anxiety-related symptoms by maintaining intestinal microbiota homeostasis or restoring intestinal microbiota function. In two different stress models, we observed that the changes in the community structure of the gut microbiota are different, such as the diversity level of the gut microbiota and differences in the gut microbiota at the phylum and genus levels, which may be due to gender differences in mice and differences in stress models. This requires us to conduct in-depth research on this issue by using mice of different genders in the same stress model in future studies.

Excellent probiotic strains would increase the product’s value and foster innovation in the development of probiotic products. Based on our research, strain WLR01 can alleviate symptoms associated with anxiety-like behavior, reduce visceral hypersensitivity, and improve cognition and memory. Such probiotics can provide comprehensive health benefits for a variety of health issues and be applied to a broader range of individuals and health conditions. However, it should be noted that this study was performed with mouse models, and the probiotic effects should be tested with clinical trials.

## 5. Conclusions

In this study, we obtained 11 strains of *Lm. reuteri*, along with their whole-genome sequences and physiological features. Our findings revealed significant genomic and phenotypic diversity among these strains, including variations in metabolism, antibiotic resistance, and probiotic properties.

Strain WLR01 notably alleviated anxiety-like behaviors in mice, improved cognitive function, and modulated gut microbiota and gastrointestinal symptoms, potentially through the NLRP3 inflammasome and HPA axis pathways. These results highlight the strong potential of strain WLR01 in treating anxiety and related symptoms, demonstrating its effectiveness in modulating physiological and behavioral responses associated with chronic stress.

## Figures and Tables

**Table 1:** The sequence of primers used for RT-qPCR.

Gene	Forward Primer (5′→3′)	Reverse Primer (5′→3′)
GAPDH	CATCACTGCCACCCAGAAGACTG	ATGCCAGTGAGCTTCCCGTTCAG
ZO-1	GTTGGTACGGTGCCCTGAAAGA	GCTGACAGGTAGGACAGACGAT
Occludin	TGGCAAGCGATCATACCCAGAG	CTGCCTGAAGTCATCCACACTC

## Data Availability

The genomic data in this study were deposited in the National Microbiology Data Center with accession number NMDC10019034 (https://nmdc.cn/resource/genomics/project/detail/NMDC10019034, accessed on 25 July 2024).
